# Supplementary material for: Wnt5a–Vangl1/2 signaling regulates the position and direction of lung branching through the cytoskeleton and focal adhesions
Source: PLoS Biol. 2022 Aug 26;20(8):e3001759. doi: 10.1371/journal.pbio.3001759 (PMC9469998; doi:10.1371/journal.pbio.3001759)
Supplement: S1 Fig — Ventral (A, B, D, E) and dorsal (C) views of dissected lungs from wild-type and Wnt5a−/− embryos at the developmental stages indicated. Ventral (F, G, I, J) and dorsal (H) views of dissected lungs from wild-type and Wnt5af/f; Sox9Cre/+ embryos at the developmental stages indicated. Ventral (K, L, N, O) and dorsal (M) views of dissected lungs from wild-type and Wnt5af/f; Dermo1Cre/+ embryos at the developmental stages indicated. (Scale bars: A-C, F-H, and K-M, 1 mm; D, E, I, J, N, and O, 1 mm.) dpc, days post coitus. (PDF) [file pbio.3001759.s001.pdf]

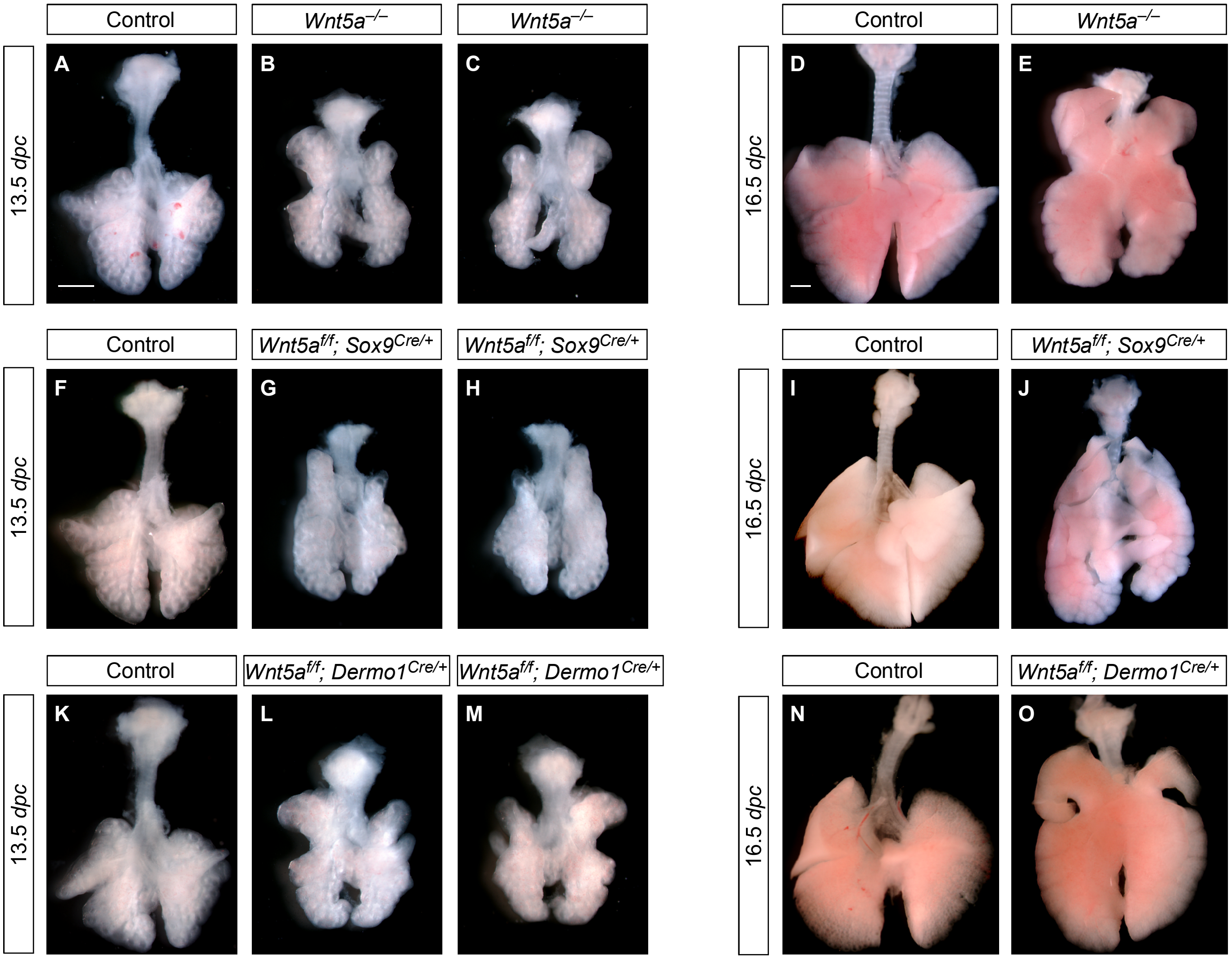

S1 Fig. Loss of *Wnt5a* leads to defective branching morphogenesis

Ventral (A, B, D, E) and dorsal (C) views of dissected lungs from wild-type and *Wnt5a*$^{-/-}$ embryos at the developmental stages indicated. Ventral (F, G, I, J) and dorsal (H) views of dissected lungs from wild-type and *Wnt5a*$^{f/f}$; *Sox9*$^{Cre/+}$ embryos at the developmental stages indicated. Ventral (K, L, N, O) and dorsal (M) views of dissected lungs from wild-type and *Wnt5a*$^{f/f}$; *Dermo1*$^{Cre/+}$ embryos at the developmental stages indicated. *dpc*, *days post coitus*. (Scale bars: A-C, F-H and K-M, 1 mm; D, E, I, J, N and O, 1 mm.)
