# Supplementary material for: Wnt5a–Vangl1/2 signaling regulates the position and direction of lung branching through the cytoskeleton and focal adhesions
Source: PLoS Biol. 2022 Aug 26;20(8):e3001759. doi: 10.1371/journal.pbio.3001759 (PMC9469998; doi:10.1371/journal.pbio.3001759)
Supplement: S2 Fig — (A-F) Immunostaining of lung sections collected from control and Wnt5af/f; Sox9Cre/+ mice at 13.5 dpc. (G) Quantification of the cell proliferation rate in the epithelium of control and Wnt5af/f; Sox9Cre/+ lungs at 13.5 dpc (mean value ± SEM, unpaired Student’s t-test, n = 3 pairs). The rate of epithelial cell proliferation was calculated as the ratio of the number of EdU+ epithelial cells (EdU+E-Cad+) to the number of epithelial cells (E-Cad+). (H-M) Immunostaining of lung sections collected from control and Wnt5af/f; Dermo1Cre/+ mice at 12.5 dpc. (N) Quantification of the cell proliferation rate in the epithelium of control and Wnt5af/f; Dermo1Cre/+ lungs at 12.5 dpc (mean value ± SEM, unpaired Student’s t-test, n = 3 pairs). The underlying data for S2G and S2N Fig and the exact P values can be found in S1 Data. (Scale bars: A-F and H-M, 25 μm.) dpc, days post coitus; ns, not significant. (PDF) [file pbio.3001759.s002.pdf]

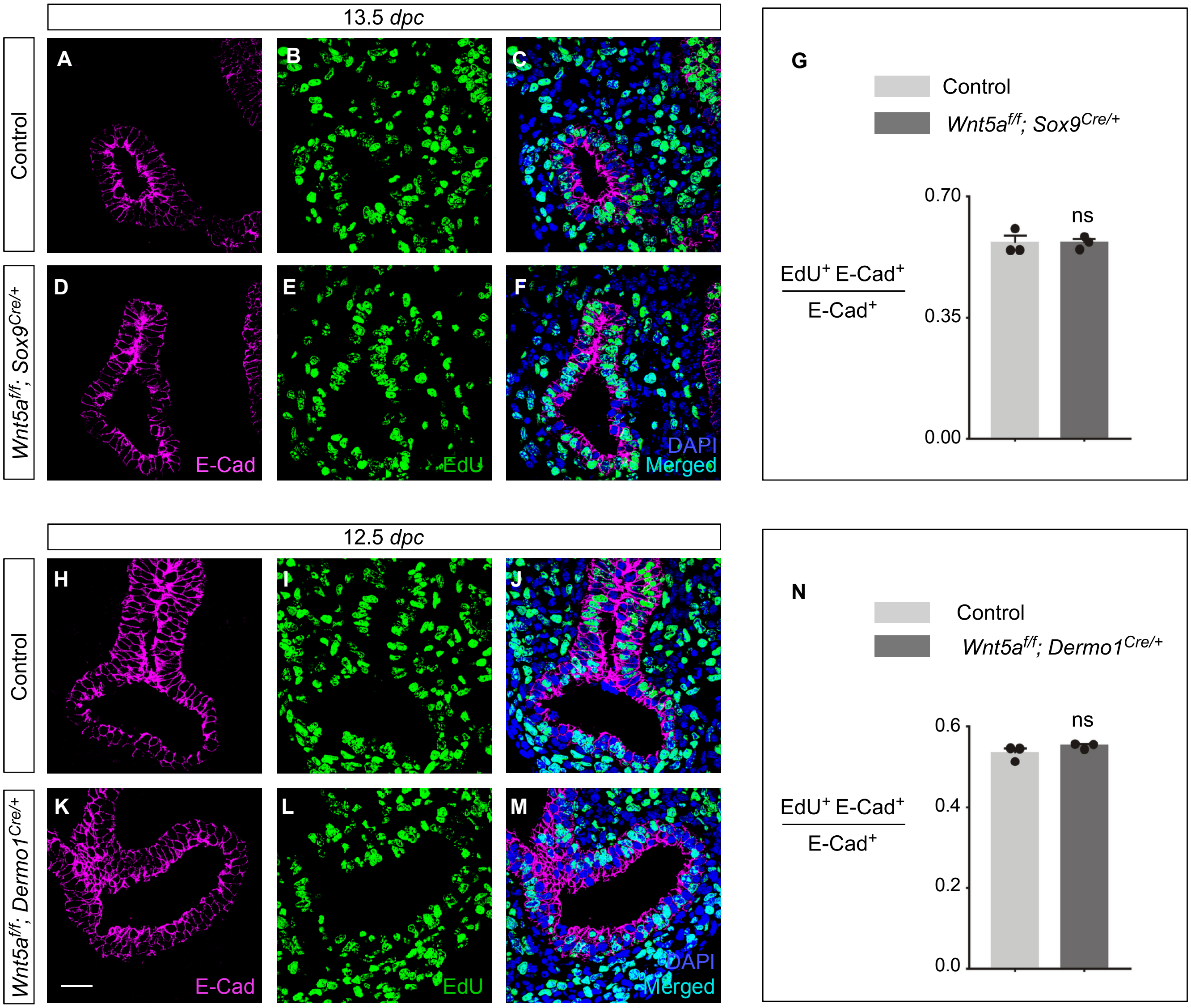

S2 Fig. The rate of cell proliferation is unaltered in the absence of *Wnt5a*

(A-F) Immunostaining of lung sections collected from control and *Wnt5a*$^{f/f}$*; Sox9*$^{Cre/+}$ mice at 13.5 *days post coitus* (*dpc*). (G) Quantification of the cell proliferation rate in the epithelium of control and *Wnt5a*$^{f/f}$*; Sox9*$^{Cre/+}$ lungs at 13.5 *dpc* (mean value ± SEM, unpaired Student's *t*-test, n = 3 pairs). The rate of epithelial cell proliferation was calculated as the ratio of the number of EdU$^+$ epithelial cells (EdU$^+$E-Cad$^+$) to the number of epithelial cells (E-Cad$^+$). (H-M) Immunostaining of lung sections collected from control and *Wnt5a*$^{f/f}$*; Dermo1*$^{Cre/+}$ mice at 12.5 *dpc*. (N) Quantification of the cell proliferation rate in the epithelium of control and *Wnt5a*$^{f/f}$*; Dermo1*$^{Cre/+}$ lungs at 12.5 *dpc* (mean value ± SEM, unpaired Student's *t*-test, n = 3 pairs). ns, not significant. The underlying data for S2G and S2N Fig, and the exact P values can be found in S1 Data. (Scale bars: A-F and H-M, 25 μm.)
