# Supplementary material for: Wnt5a–Vangl1/2 signaling regulates the position and direction of lung branching through the cytoskeleton and focal adhesions
Source: PLoS Biol. 2022 Aug 26;20(8):e3001759. doi: 10.1371/journal.pbio.3001759 (PMC9469998; doi:10.1371/journal.pbio.3001759)
Supplement: S3 Fig — Ventral (A, B, D, E, G, H) and dorsal (C, F, I) views of dissected lungs from wild-type and Vangl1gt/gt; Vangl2−/− embryos at the developmental stages indicated. (Scale bars: A-C, 0.5 mm; D-F, 1 mm; G-I, 1 mm.) (PDF) [file pbio.3001759.s003.pdf]

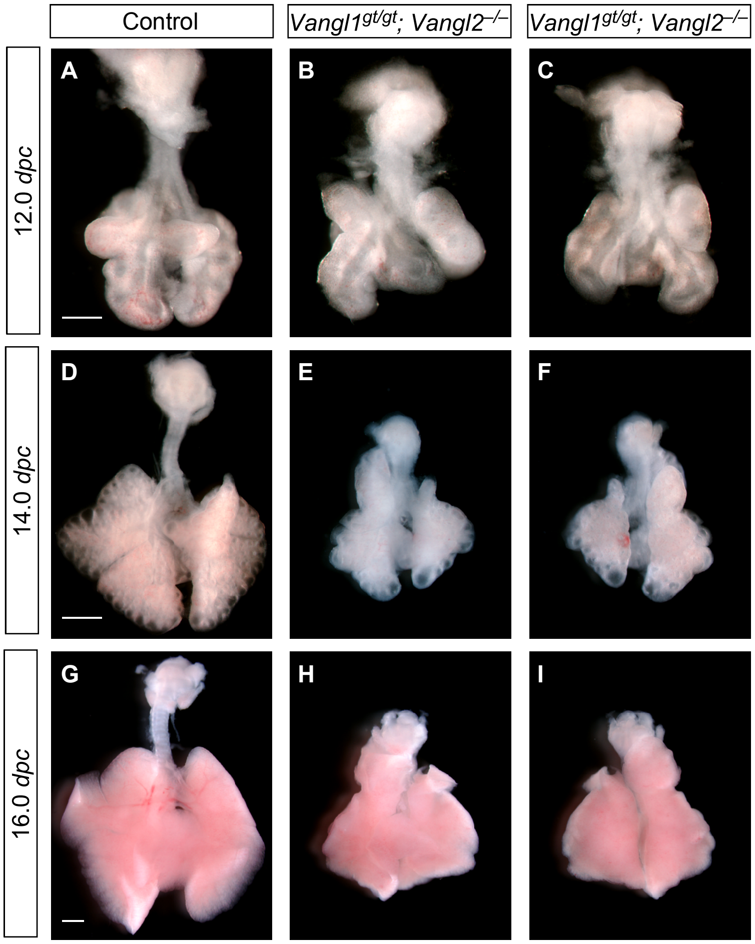

S3 Fig. *Vangl1/2* control the position and direction of lung branching
Ventral (A, B, D, E, G, H) and dorsal (C, F, I) views of dissected lungs from wild-type and *Vangl1*$^{gt/gt}$; *Vangl2*$^{-/-}$ embryos at the developmental stages indicated. (Scale bars: A-C, 0.5 mm; D-F, 1 mm; G-I, 1 mm.)
