# Supplementary material for: Wnt5a–Vangl1/2 signaling regulates the position and direction of lung branching through the cytoskeleton and focal adhesions
Source: PLoS Biol. 2022 Aug 26;20(8):e3001759. doi: 10.1371/journal.pbio.3001759 (PMC9469998; doi:10.1371/journal.pbio.3001759)
Supplement: S4 Fig — (A-D) Immunostaining of lung sections collected from control and Vangl1gt/gt; Vangl2−/− mice at 15.5 dpc. SMA marks smooth muscle cells; CD31 labels endothelial cells. (Scale bar: A-D, 25 μm.) dpc, days post coitus. (PDF) [file pbio.3001759.s004.pdf]

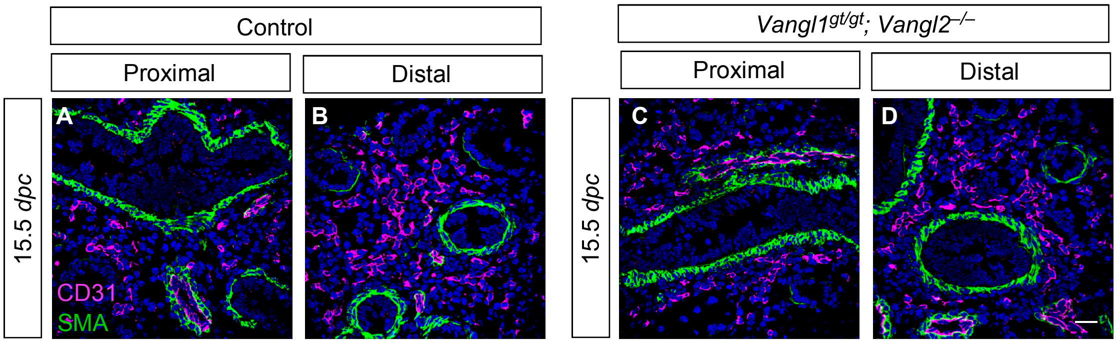

S4 Fig. Smooth muscle cells and blood vessels are unaffected in *Vangl1/2* mutant lungs
(A-D) Immunostaining of lung sections collected from control and *Vangl1*$^{gt/gt}$; *Vangl2*$^{-/-}$ mice at 15.5 *days post coitus* (*dpc*). SMA marks smooth muscle cells; CD31 labels endothelial cells. (Scale bar: A-D, 25 μm.)
