# Supplementary material for: Wnt5a–Vangl1/2 signaling regulates the position and direction of lung branching through the cytoskeleton and focal adhesions
Source: PLoS Biol. 2022 Aug 26;20(8):e3001759. doi: 10.1371/journal.pbio.3001759 (PMC9469998; doi:10.1371/journal.pbio.3001759)
Supplement: S5 Fig — (A-F) Immunostaining of lung sections collected from control and Wnt5a−/− mice at 12.5 dpc. (G) Quantification of VANGL2 signal in lung cells of control or Wnt5a mutant lungs (mean value ± SEM, unpaired Student’s t-test, n = 4 pairs). (H-M) Immunostaining of lung sections collected from control and Vangl2f/f; Sox9Cre/+ lungs at 14.5 dpc. (N-Q) Ventral views of dissected lungs from control and mutant lungs at 14.5 dpc. The underlying data for S5G Fig and the exact P value can be found in S1 Data. (Scale bar: A-F, H-M, 25 μm; N-Q, 1 mm) dpc, days post coitus; ns, not significant. (PDF) [file pbio.3001759.s005.pdf]

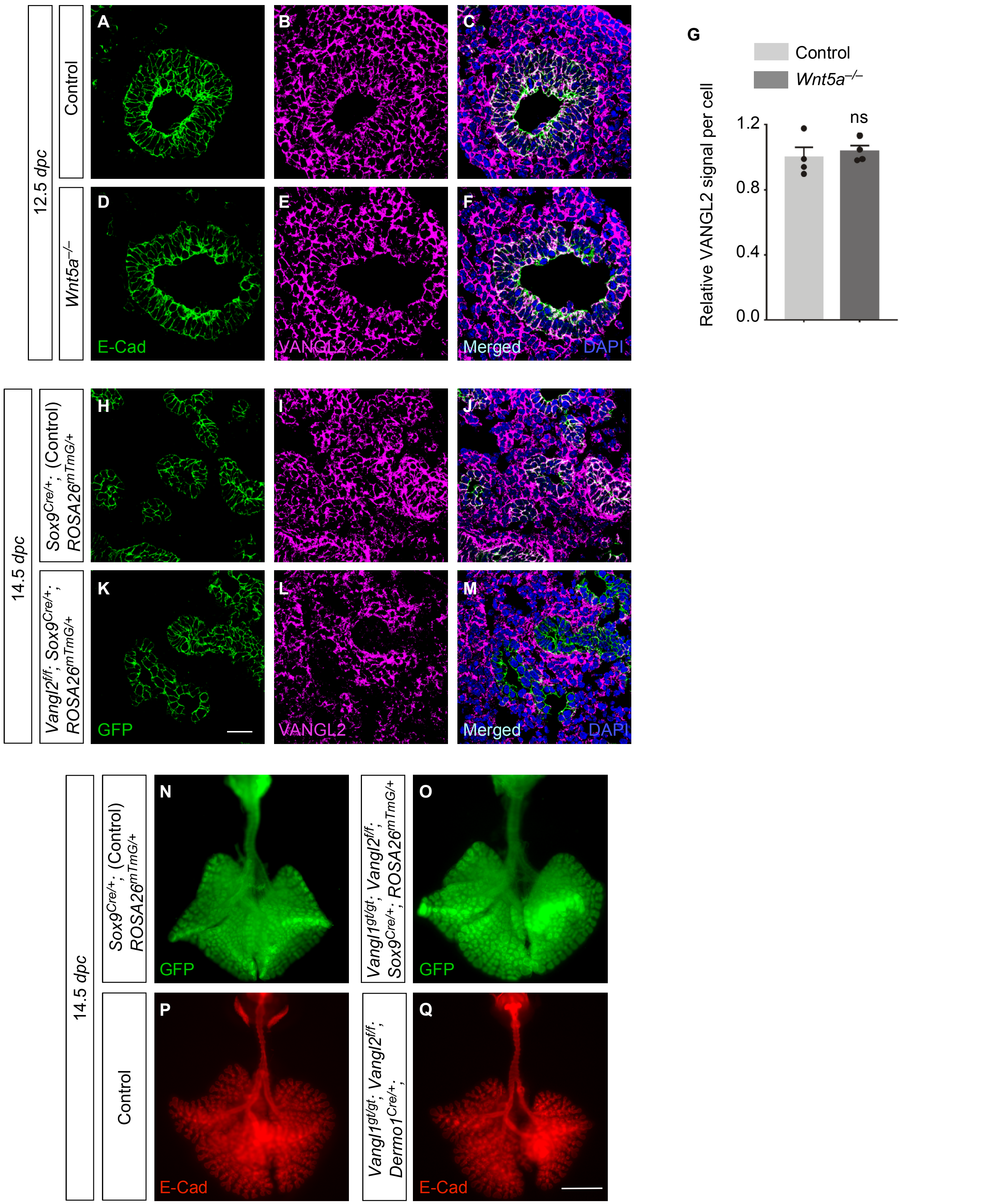

S5 Fig. Selective loss of *Vangl1/2* in the lung epithelium or mesenchyme does not lead to branching defects

(A-F) Immunostaining of lung sections collected from control and *Wnt5a*$^{-/-}$ mice at 12.5 *days post coitus* (*dpc*). (G) Quantification of VANGL2 signal in lung cells of control or *Wnt5a* mutant lungs (mean value ± SEM, unpaired Student's *t*-test, n = 4 pairs). (H-M) Immunostaining of lung sections collected from control and *Vangl2*$^{f/f}$*; Sox9*$^{Cre/+}$ lungs at 14.5 *dpc*. (N-Q) Ventral views of dissected lungs from control and mutant lungs at 14.5 *dpc*. ns, not significant. The underlying data for S5G Fig and the exact P value can be found in S1 Data. (Scale bar: A-F, H-M, 25 μm; N-Q, 1 mm)
