# Supplementary material for: Wnt5a–Vangl1/2 signaling regulates the position and direction of lung branching through the cytoskeleton and focal adhesions
Source: PLoS Biol. 2022 Aug 26;20(8):e3001759. doi: 10.1371/journal.pbio.3001759 (PMC9469998; doi:10.1371/journal.pbio.3001759)
Supplement: S6 Fig — (A) qPCR analysis of Foxa2 transcript levels in control and Wnt5a−/− lungs at 12.5 and 13.5 dpc (mean value ± SEM, unpaired Student’s t-test, n = 3 pairs). (B) qPCR analysis of Foxa2 transcript levels in control and Vangl1gt/gt; Vangl2−/− lungs at 12.5 and 13.5 dpc (mean value ± SEM, unpaired Student’s t-test, n = 3 pairs). (C) qPCR analysis of components of the major signaling pathways in control and Wnt5a−/− lungs at 12.5 dpc (mean value ± SEM, unpaired Student’s t-test, n = 3 pairs). (*) p < 0.05; (**) p < 0.01. The underlying data for S5A–S5C Fig and the exact P values can be found in S1 Data. dpc, days post coitus; ns, not significant. (PDF) [file pbio.3001759.s006.pdf]

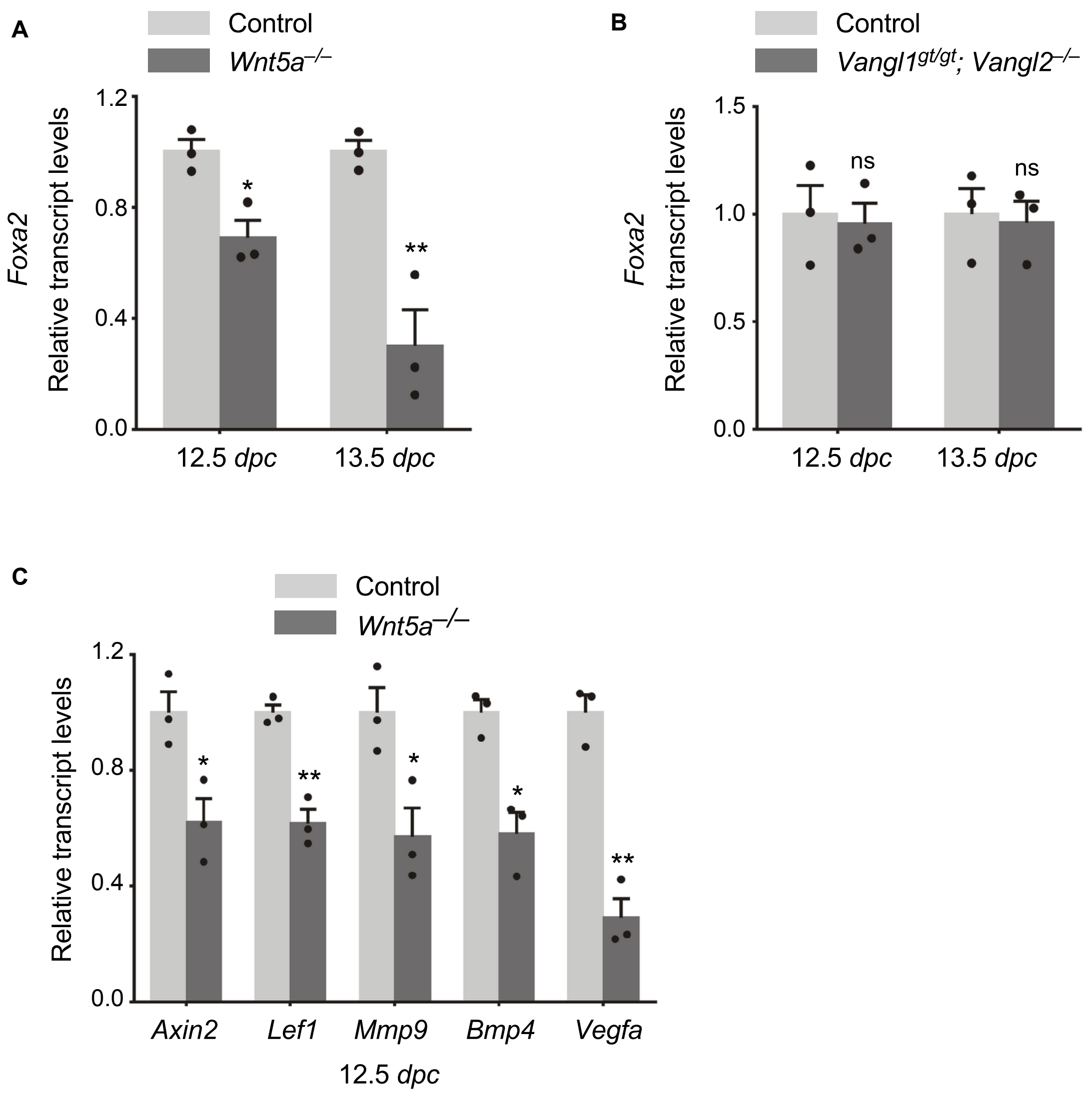

S6 Fig. The transcription factor, *Foxa2*, and major signaling pathways in the developing lungs are perturbed in the absence of *Wnt5a*

(A) qPCR analysis of *Foxa2* transcript levels in control and *Wnt5a*$^{-/-}$ lungs at 12.5 and 13.5 *days post coitus* (*dpc*) (mean value ± SEM, unpaired Student's *t*-test, n = 3 pairs). (B) qPCR analysis of *Foxa2* transcript levels in control and *Vangl1*$^{gt/gt}$*; Vangl2*$^{-/-}$ lungs at 12.5 and 13.5 *dpc* (mean value ± SEM, unpaired Student's *t*-test, n = 3 pairs). (C) qPCR analysis of components of the major signaling pathways in control and *Wnt5a*$^{-/-}$ lungs at 12.5 *dpc* (mean value ± SEM, unpaired Student's *t*-test, n = 3 pairs). (*) $p<0.05$; (**) $p<0.01$; ns, not significant. The underlying data for S6A, S6B and S6C Fig, and the exact P values can be found in S1 Data.
