# Supplementary material for: Wnt5a–Vangl1/2 signaling regulates the position and direction of lung branching through the cytoskeleton and focal adhesions
Source: PLoS Biol. 2022 Aug 26;20(8):e3001759. doi: 10.1371/journal.pbio.3001759 (PMC9469998; doi:10.1371/journal.pbio.3001759)
Supplement: S7 Fig — (A-L) Whole-mount immunostaining of dissected lungs from control and Wnt5a−/− mice at 12.5 dpc. Lung epithelium was visualized by E-cadherin (E-Cad). Circles in (H, K) indicate defective branching in Wnt5a-deficient lungs. (M-T) Immunostaining of lung sections collected from control and Wnt5a−/− mice at 12.5 dpc. (Scale bars: A-L, 0.5 mm; M-T, 25 μm.) dpc, days post coitus; p-FAK, phosphorylated FAK. (PDF) [file pbio.3001759.s007.pdf]

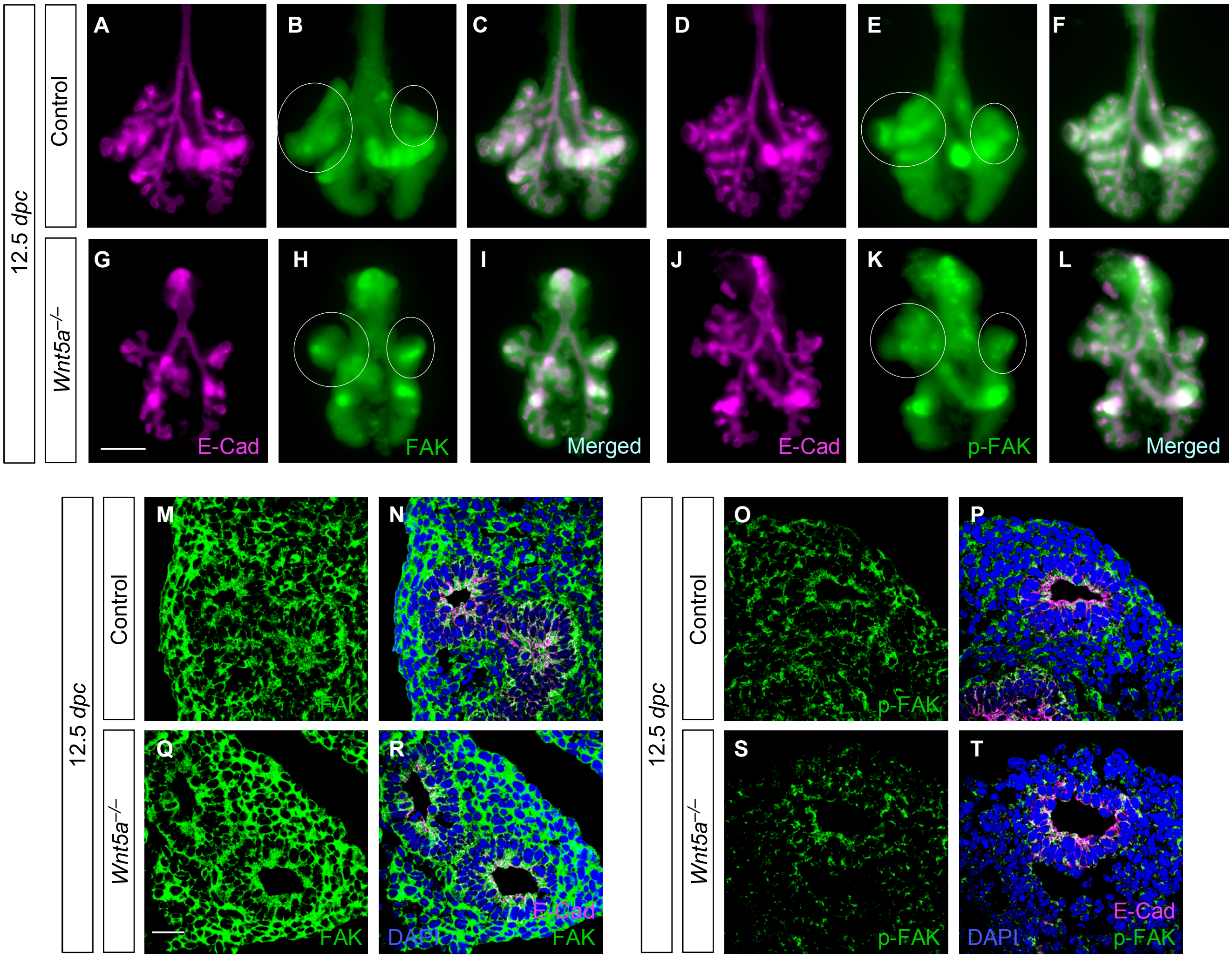

S7 Fig. Phosphorylated FAK (p-FAK) is reduced in *Wnt5a*-null lungs

(A-L) Whole-mount immunostaining of dissected lungs from control and *Wnt5a*$^{-/-}$ mice at 12.5 *days post coitus* (*dpc*). Lung epithelium was visualized by E-cadherin (E-Cad). Circles in (H, K) indicate defective branching in *Wnt5a*-deficient lungs. (M-T) Immunostaining of lung sections collected from control and *Wnt5a*$^{-/-}$ mice at 12.5 *dpc*. (Scale bars: A-L, 0.5 mm; M-T, 25 μm.)
