# Supplementary material for: Wnt5a–Vangl1/2 signaling regulates the position and direction of lung branching through the cytoskeleton and focal adhesions
Source: PLoS Biol. 2022 Aug 26;20(8):e3001759. doi: 10.1371/journal.pbio.3001759 (PMC9469998; doi:10.1371/journal.pbio.3001759)
Supplement: S8 Fig — (A-L) Immunostaining of lung sections collected from control and Wnt5af/f; Sox9Cre/+ lungs at 13.5 dpc. Lung epithelium was visualized by E-cadherin (E-Cad). (M-X) Immunostaining of lung sections collected from control and Wnt5af/f; Dermo1Cre/+ lungs at 12.5 dpc. (Scale bar: A-X, 25 μm.) dpc, days post coitus; p-FAK, phosphorylated FAK. (PDF) [file pbio.3001759.s008.pdf]

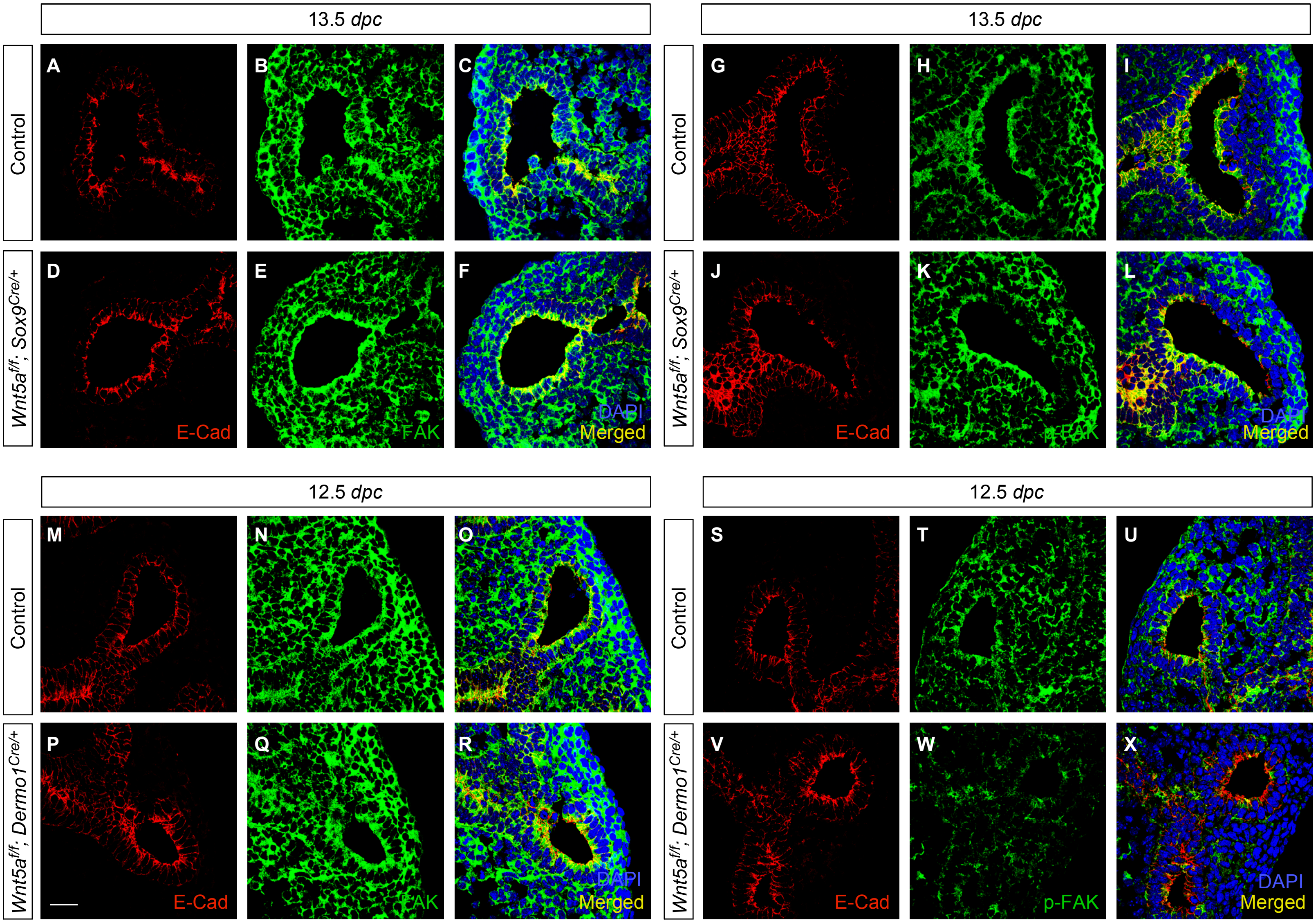

S8 Fig. Phosphorylated FAK (p-FAK) is reduced in *Wnt5a*-deficient lung compartments
(A-L) Immunostaining of lung sections collected from control and *Wnt5a*$^{f/f}$*; Sox9*$^{Cre/+}$ lungs at 13.5 *days post coitus* (*dpc*). Lung epithelium was visualized by E-cadherin (E-Cad). (M-X) Immunostaining of lung sections collected from control and *Wnt5a*$^{f/f}$*; Dermo1*$^{Cre/+}$ lungs at 12.5 *dpc*. (Scale bar: A-X, 25 μm.)
