# Supplementary material for: Wnt5a–Vangl1/2 signaling regulates the position and direction of lung branching through the cytoskeleton and focal adhesions
Source: PLoS Biol. 2022 Aug 26;20(8):e3001759. doi: 10.1371/journal.pbio.3001759 (PMC9469998; doi:10.1371/journal.pbio.3001759)
Supplement: S9 Fig — (A) Western blot analysis of cell lysates derived from control and Wnt5a−/− lungs at 12.5 dpc. (B) Western blot analysis of cell lysates derived from control and Vangl1gt/gt; Vangl2−/− lungs at 12.5 dpc. (C) Western blot analysis of cell lysates derived from control cells, Vangl1gt/gt; Vangl2−/− cells and Vangl1gt/gt; Vangl2−/− cells expressing VANGL2 or VANGL2 (84A) as indicated. α-tubulin serves as the loading control. dpc, days post coitus; FAK, focal adhesion kinase; p-FAK, phosphorylated FAK. (PDF) [file pbio.3001759.s009.pdf]

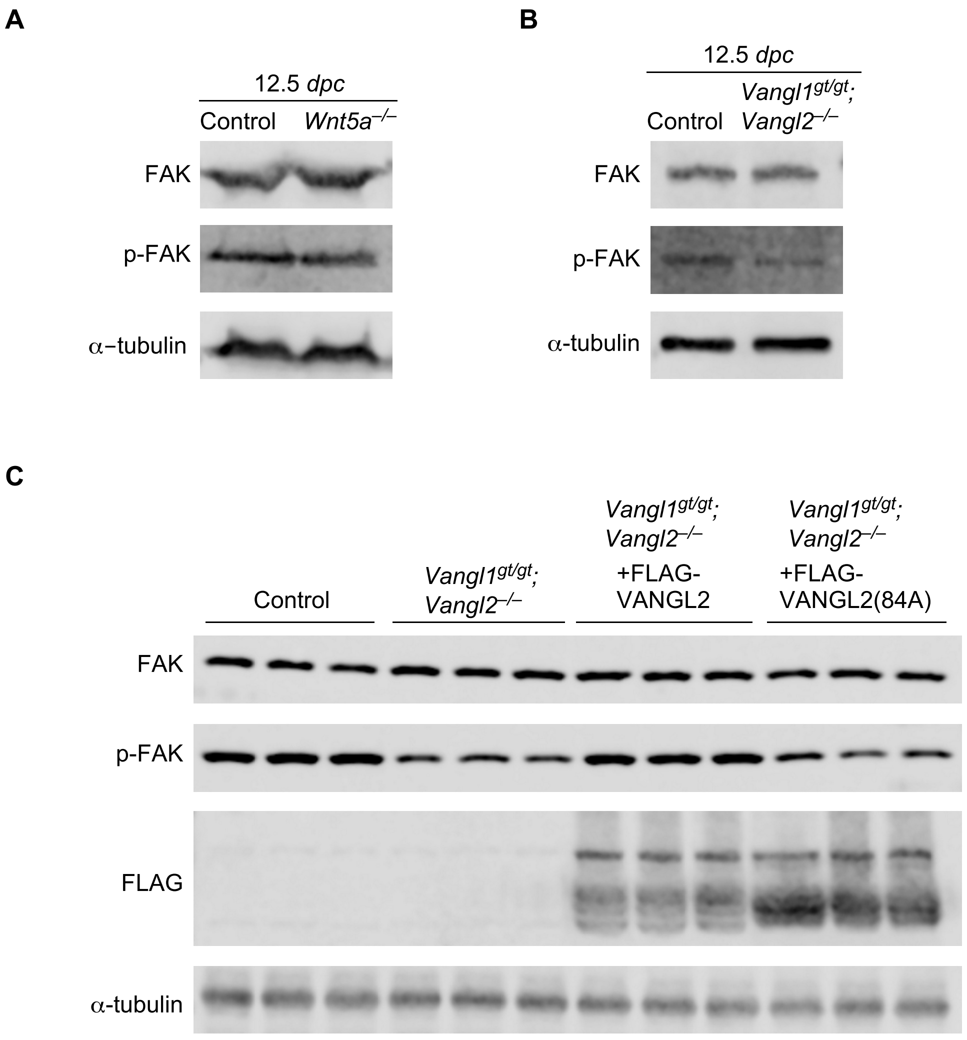

S9 Fig. Phosphorylated FAK (p-FAK) is reduced in the absence of *Wnt5a* or *Vangl1/2*

(A) Western blot analysis of cell lysates derived from control and *Wnt5a*$^{-/-}$ lungs at 12.5 *days post coitus* (*dpc*). (B) Western blot analysis of cell lysates derived from control and *Vangl1*$^{gt/gt}$; *Vangl2*$^{-/-}$ lungs at 12.5 *dpc*. (C) Western blot analysis of cell lysates derived from control cells, *Vangl1*$^{gt/gt}$; *Vangl2*$^{-/-}$ cells and *Vangl1*$^{gt/gt}$; *Vangl2*$^{-/-}$ cells expressing VANGL2 or VANGL2 (84A) as indicated. α-tubulin serves as the loading control.
