# Supplementary material for: Wnt5a–Vangl1/2 signaling regulates the position and direction of lung branching through the cytoskeleton and focal adhesions
Source: PLoS Biol. 2022 Aug 26;20(8):e3001759. doi: 10.1371/journal.pbio.3001759 (PMC9469998; doi:10.1371/journal.pbio.3001759)
Supplement: S10 Fig — (A-L) Immunostaining of lung sections collected from control and Vangl1gt/gt; Vangl2−/− mice at 12.5 dpc. Lung epithelium was marked by E-cadherin (E-Cad). (M) Quantification of the relative density of Cofilin and p-Cofilin in lung cells at the epithelial tip or in the mesenchyme (mean value ± SEM, unpaired Student’s t-test, n = 4 pairs). (**) p < 0.01. The underlying data for S10M Fig and the exact P values can be found in S1 Data. (Scale bar: A-L, 25 μm.) dpc, days post coitus; ns, not significant; p-Cofilin, phosphorylated Cofilin; (PDF) [file pbio.3001759.s010.pdf]

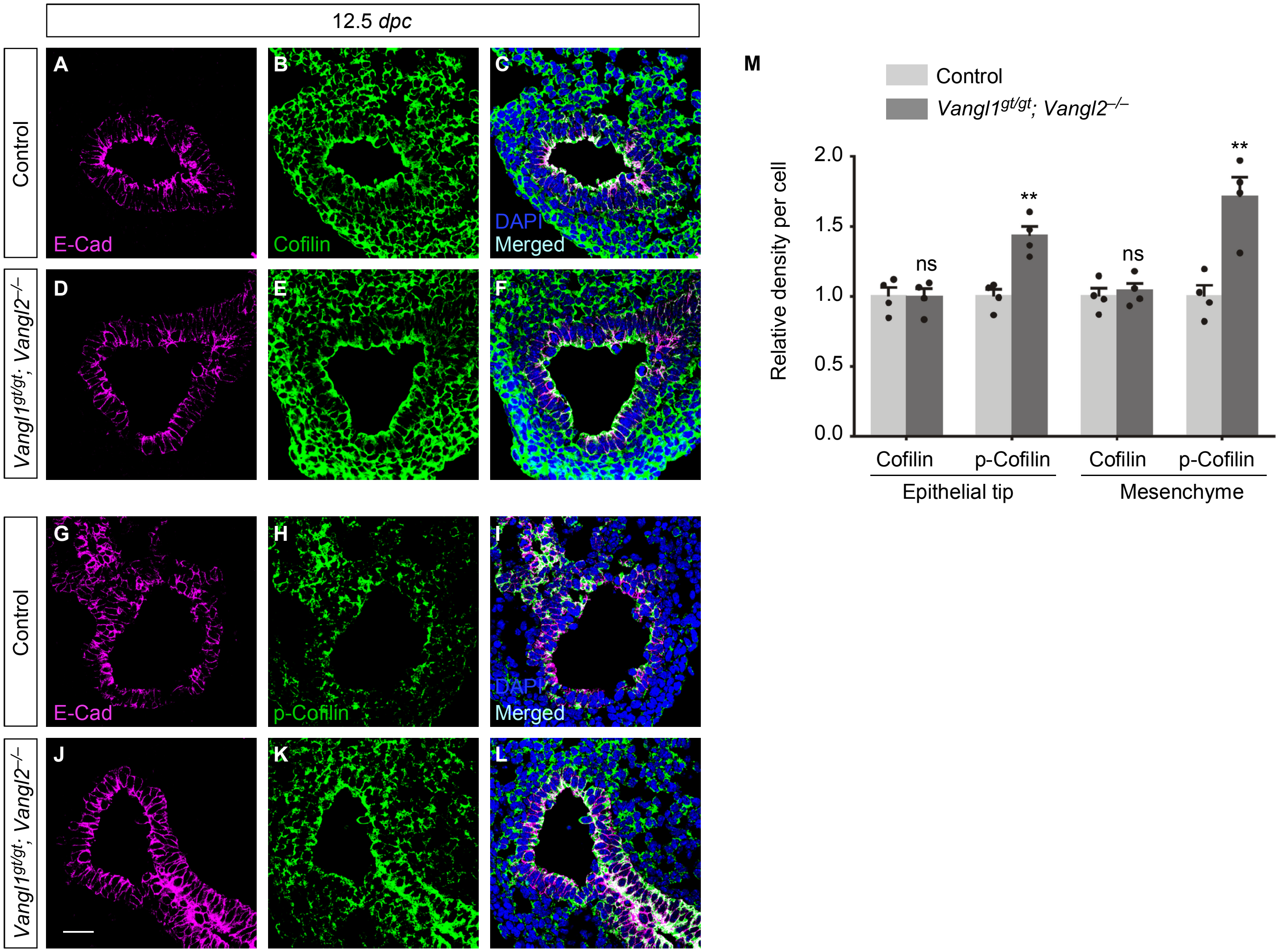

S10 Fig. Phosphorylated Cofilin (p-Cofilin) levels are increased in the absence of *Vangl1/2*

(A-L) Immunostaining of lung sections collected from control and *Vangl1*$^{gt/gt}$; *Vangl2*$^{-/-}$ mice at 12.5 *days post coitus* (*dpc*). Lung epithelium was marked by E-cadherin (E-Cad). (M) Quantification of the relative density of Cofilin and p-Cofilin in lung cells at the epithelial tip or in the mesenchyme (mean value ± SEM, unpaired Student's *t*-test, n = 4 pairs). (**) p<0.01; ns, not significant. The underlying data for S10M Fig and the exact P values can be found in S1 Data. (Scale bar: A-L, 25 μm.)
