# Supplementary material for: Wnt5a–Vangl1/2 signaling regulates the position and direction of lung branching through the cytoskeleton and focal adhesions
Source: PLoS Biol. 2022 Aug 26;20(8):e3001759. doi: 10.1371/journal.pbio.3001759 (PMC9469998; doi:10.1371/journal.pbio.3001759)
Supplement: S11 Fig — (A-X) Immunostaining of lung sections collected from control and Vangl1gt/gt; Vangl2−/− mice at 12.5 dpc. Lung epithelium was marked by E-cadherin (E-Cad). F-actin was labeled by phalloidin. (Y) Quantification of phalloidin signal in lung cells of control or Vangl1/2 mutant lungs (mean value ± SEM, unpaired Student’s t-test, n = 4 pairs). (Z) Quantification of pMLC signal in lung cells of control or Vangl1/2 mutant lungs (mean value ± SEM, unpaired Student’s t-test, n = 4 pairs). The underlying data for S11Y and S11Z Fig and the exact P values can be found in S1 Data. (Scale bar: A-X, 25 μm.) dpc, days post coitus; ns, not significant; pMLC, phosphorylated myosin light chain. (PDF) [file pbio.3001759.s011.pdf]

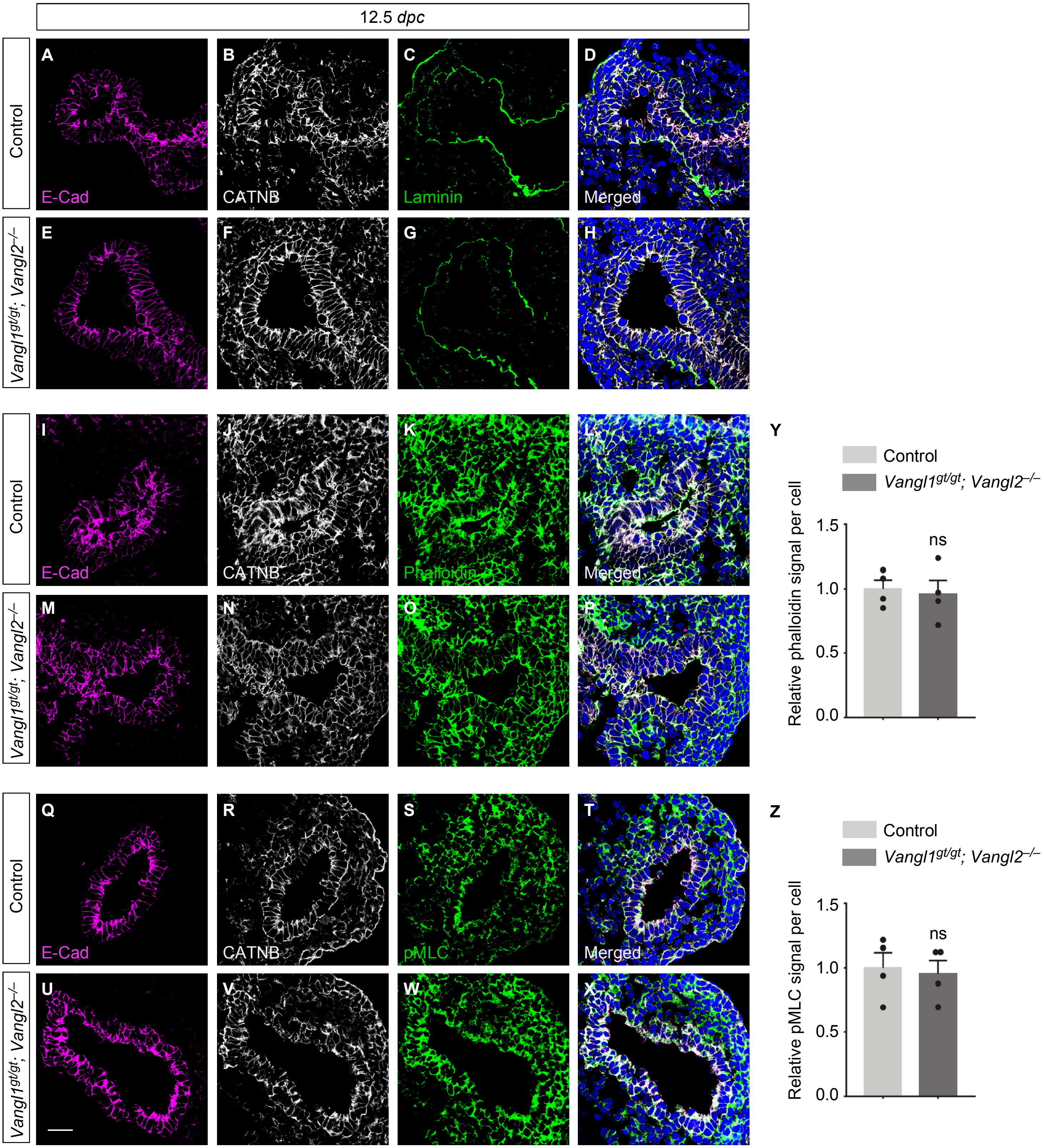

S11 Fig. Expression level of Laminin, F-actin and phosphorylated myosin light chain (pMLC) are unaltered in *Vangl1/2* mutant lungs

(A-X) Immunostaining of lung sections collected from control and *Vangl1*$^{gt/gt}$; *Vangl2*$^{-/-}$ mice at 12.5 *days post coitus* (*dpc*). Lung epithelium was marked by E-cadherin (E-Cad). F-actin was labeled by phalloidin. (Y) Quantification of phalloidin signal in lung cells of control or *Vangl1/2* mutant lungs (mean value ± SEM, unpaired Student's *t*-test, n = 4 pairs). (Z) Quantification of pMLC signal in lung cells of control or *Vangl1/2* mutant lungs (mean value ± SEM, unpaired Student's *t*-test, n = 4 pairs). ns, not significant. The underlying data for S11Y and S11Z Fig, and the exact P values can be found in S1 Data. (Scale bar: A-X, 25 μm.)
