# Supplementary material for: Wnt5a–Vangl1/2 signaling regulates the position and direction of lung branching through the cytoskeleton and focal adhesions
Source: PLoS Biol. 2022 Aug 26;20(8):e3001759. doi: 10.1371/journal.pbio.3001759 (PMC9469998; doi:10.1371/journal.pbio.3001759)
Supplement: S12 Fig — (A-X) Immunostaining of lung explants treated with control media or media containing 0.5 μM or 2 μM of PF-573228 (FAK inhibitor) as indicated. (Y) Quantification of the relative density of FAK and p-FAK in lung cells treated with PF-573228 (mean value ± SEM, one-way ANOVA, n = 5 pairs). (*) p < 0.05; (**) p < 0.01; (***) p < 0.001. The underlying data for S12Y Fig can be found in S1 Data. (Scale bar: A-X, 25 μm.) FAK, focal adhesion kinase; ns, not significant; p-FAK, phosphorylated FAK. (PDF) [file pbio.3001759.s012.pdf]

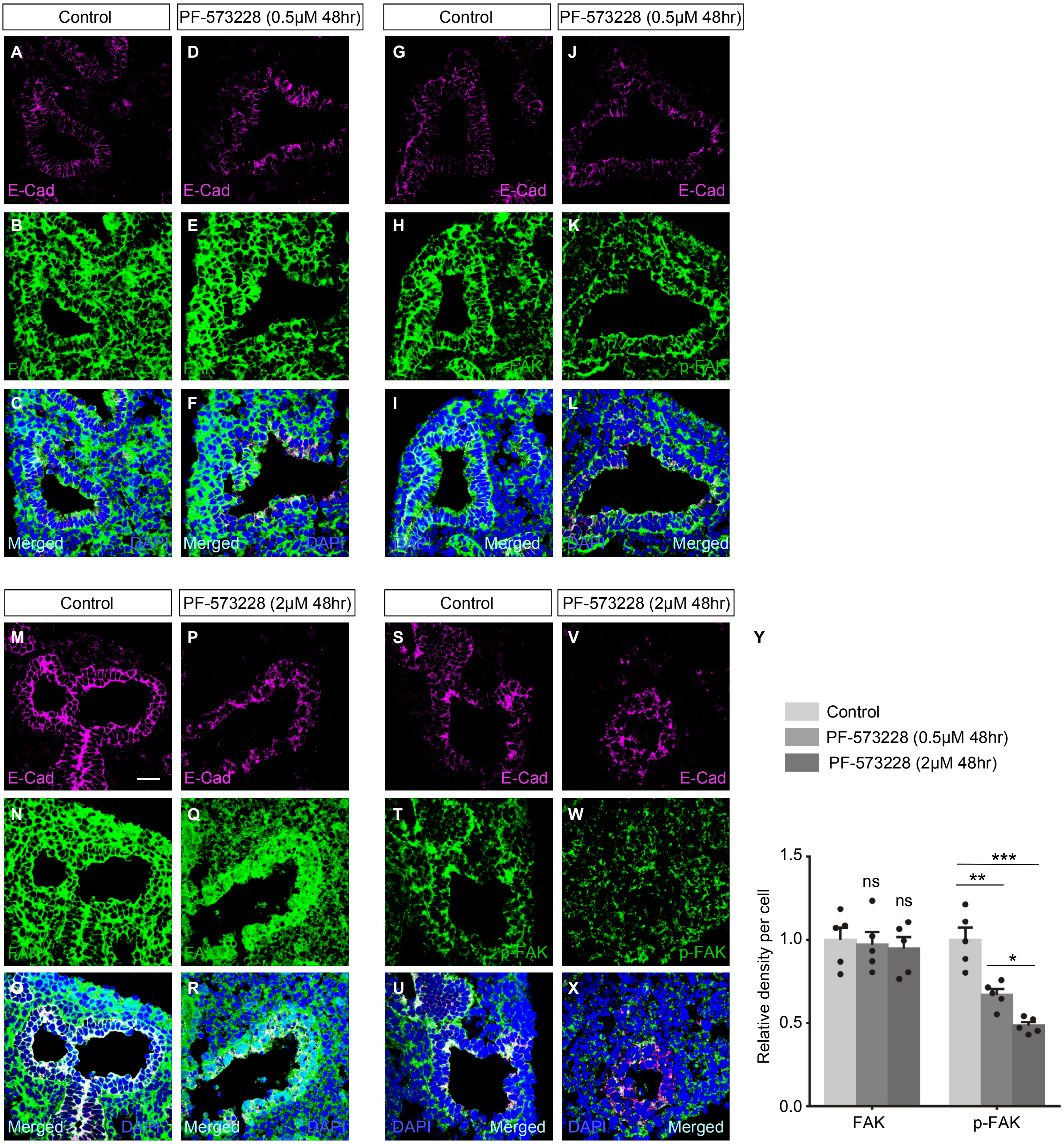

S12 Fig. Phosphorylated FAK (p-FAK) is reduced in the presence of FAK inhibitor
(A-X) Immunostaining of lung explants treated with control media or media containing 0.5 μM or 2 μM of PF-573228 (FAK inhibitor) as indicated. (Y) Quantification of the relative density of FAK and p-FAK in lung cells treated with PF-573228 (mean value ± SEM, One-Way ANOVA, n = 5 pairs). (*) p<0.05; (**) p<0.01; (***) p<0.001; ns, not significant. The underlying data for S12Y Fig can be found in S1 Data. (Scale bar: A-X, 25 μm.)
