# Supplementary material for: Wnt5a–Vangl1/2 signaling regulates the position and direction of lung branching through the cytoskeleton and focal adhesions
Source: PLoS Biol. 2022 Aug 26;20(8):e3001759. doi: 10.1371/journal.pbio.3001759 (PMC9469998; doi:10.1371/journal.pbio.3001759)

1 Control lungs
2 *Wnt5a* KO lungs

3 Control lungs
4 *Vangl1/2* KO lungs

5 Control cells
6 *Vangl1/2* KO cells
7 *Vangl1/2* KO cells + FLAG-VANGL2
8 *Vangl1/2* KO cells + FLAG-VANGL2 (84A)

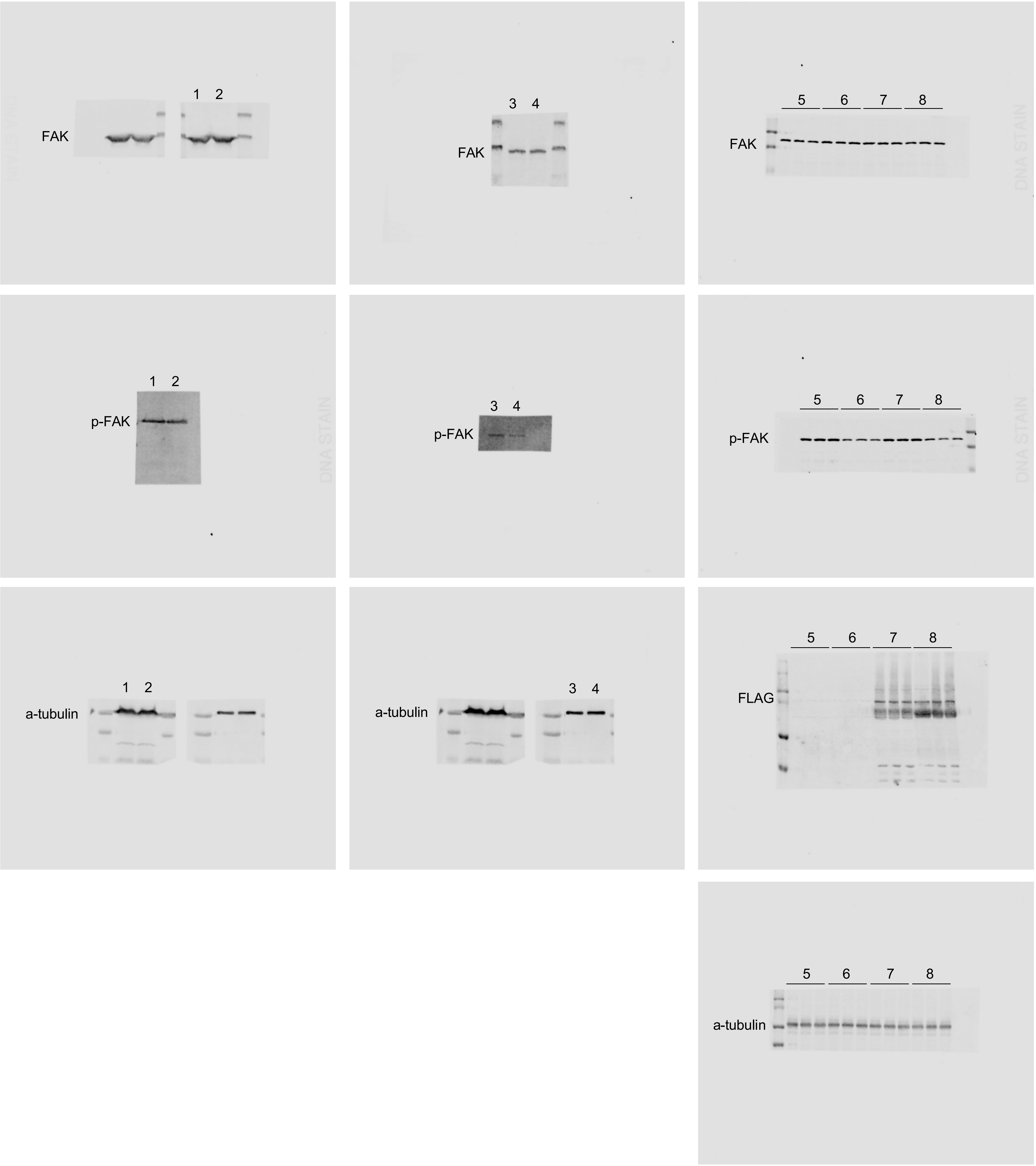

Supplement: S1 Raw Images — (PDF) [file pbio.3001759.s013.pdf]
