# Supplementary material for: Wnt5a–Vangl1/2 signaling regulates the position and direction of lung branching through the cytoskeleton and focal adhesions
Source: PLoS Biol. 2022 Aug 26;20(8):e3001759. doi: 10.1371/journal.pbio.3001759 (PMC9469998; doi:10.1371/journal.pbio.3001759)
Supplement: Attachment — Submitted filename: Response to Reviewers.pdf [file pbio.3001759.s016.pdf]

Rev. 1:
In this manuscript from the lab of Pao-Tien Chuang, the authors report a very interesting lung branch positioning defect in Wnt5a knockout, mesenchymal Wnt5a deletion, epithelial Wnt5a deletion, and Vangl1/2 knockout mice, beginning around E11.5. They also report that subsequent branching defects were more severe in Wn5a deletion than with Vangl1/2 deletion, from which they conclude that Wnt5a has additional effects on canonical Wnt signaling including driving expression of Foxa2. Comparative bulk RNA sequencing with pathway analysis revealed perturbation in focal adhesion, ECM-receptor interactions, and actomyosin cytoskeleton, confirmed by proteomic analysis. They then localize p-FAK in wild type and mutant lungs by antibody staining and find it to be reduced in Vangl1/2 and Wnt5a mutant lungs, while proliferation is no different. They also use an FAK inhibitor in lung explant cultures which results in branch size and position defects. They propose that the Wnt5a-Vangl1/2 signaling axis regulates the position and direction of lung branching by regulating the cytoskeleton and focal adhesions. While the studies are of high quality and involve state of the art in vivo gene deletion with precision measurement of branching defects, it is not convincing that their model can explain the phenotype as they claim in their manuscript title.

Major criticisms:
1) It is hard to reconcile how perturbed cytoskeletal control and focal adhesions would result in mis-localized branches. I would rather expect truncated or failed branching defects at the normal site of initiation. It seems more likely that these mechanical defects would affect execution of branch outgrowth as opposed to specification of the site of branching.

Our analysis of *Wnt5a* and *Vangl1/2* mutant lungs from 11.5 *dpc* and onward clearly showed a defect in the position and direction of branching. To further understand how *Wnt5a–Vangl/2* signaling controls this process, we traced lung development in control and *Vangl1/2* mutant lungs from 11.0–11.5 *dpc*. We found that the epithelium where RCr/RMd and L.L1/L.L2 emerge underwent coordinated morphological changes in control lungs (Fig. 2Z–2C' in the revision). The lumen was enlarged first. Rudiments of RCr/RMd and L.L1/L.L2 were then formed. Meanwhile, the mesenchyme appeared to "push down" the epithelium between the two future branches. Finally, RCr/RMd and L.L1/L.L2 emerged at the defined position and direction.

Wnt5a–Vangl1/2 signaling is known to control the cytoskeleton as shown in previous studies and focal adhesions in our current work. We thus speculate that the mechanical force between cells is affected in the absence of *Wnt5a–Vangl1//2* signaling. This could change the overall shape and orientation of the epithelial sheet. As a result, the relative position and direction of RCr/RMd and L.L1/L.L2 are affected (Fig. 2D'–2G' in the revision). A model that illustrates this notion is depicted in Fig. 4Z. We have clarified this point in the revision.

2) Since Wnt5a deletion in either mesenchyme or epithelium appears to have the same result, and since p-FAK is active in both compartments in wild type lungs and reduced in both compartments in mutant lungs and with FAK inhibitor, it is not clear whether the phenotype can be ascribed to defective cytoskeletal control in the epithelial or mesenchymal compartment. In the Wnt5a knockout lung at E12.5, it even looks like p-FAK is only depleted in the mesenchyme (Figure S5 P,T), while the model suggests epithelial loss drives the phenotype. In all stains, there still appears to be p-FAK staining present in knockout lungs, so it is not clear that its absence underlies the phenotype.

We have added data to show that removal of *Vangl1/2* in the lung epithelium by *Sox9-Cre* and in the mesenchyme by *Dermo1-Cre* does not lead to apparent branching defects (Fig. S5N–Q in the revision). This is in contrast to the branching defects observed in *Vangl1/2* null lungs. Barring the onset and efficiency of these two Cre lines, the simplest interpretation is that coordination of *Vangl1/2* signaling (hence the downstream effectors) in both the epithelium and mesenchyme contribute to lung branching.

p-FAK levels are reduced (but not absent) in both the epithelium and mesenchyme of *Wnt5a* or *Vangl1/2* mutant lungs (Fig. 3S, 3T, 3V, 3W in the revision) or in *Vangl1/2*-deficient cell lines. Moreover, lung explants treated with FAK inhibitors are associated with reduced p-FAK levels and branching defects. This suggests that p-FAK is one of the many downstream effectors of *Wnt5a–Vangl1/2* signaling and can contribute to lung branching. However, effectors other than p-FAK also contribute to lung branching. These points have been clarified in the revision.

3) There is incomplete penetrance in some genotypes which is not addressed. Perhaps the authors could examine p-FAK signaling in unaffected versus affected lungs from the same litter to see if this correlates, which would support their claim that defective focal adhesions are responsible for their phenotype.

Reduction in p-FAK levels and severity of branching in lung explants treated with FAK inhibitors correlate with the concentration of FAK inhibitors (Fig. S12 in the revision). However, it is difficult to establish such a correlation in the lungs. Nevertheless, p-FAK is reduced in all *Vangl1/2* knockout lungs even though their phenotypes are milder than those in *Wnt5a* knockout lungs. This suggests that p-FAK mediates important aspects of the phenotypes. The more severe phenotypes in *Wnt5a* mutant lungs compared to *Vangl1/2* knockout lungs could be due to additional targets of *Wnt5a*. Incomplete penetrance suggests the involvement of multiple pathways, which requires future investigations. In addition, certain branches are preferentially affected. This raises that possibility that different pathway combinations are employed at distinct locations. We have discussed this point in the revision.

4) In several important experiments, there was no statistical analysis performed (for example Figure 1, J-L and Figure 2, P-R).

We have added statistical analysis to Fig. 1J, 1K, 1L and Fig. 2P, 2Q, 2R.

Minor criticisms:
1) Data that is bi-modally distributed could be analyzed using a bi-modal statistical approach.

We have changed the way bi-modally distributed data are analyzed in Fig. 1K.

2) In some cases the wrong statistical test is applied. For example, in Figure 3T the authors should use a one-way ANOVA.

We have used one-ANOVA for determining the statistical significance between three or more independent groups, such as that in Fig. 3T (now Fig. 3C’ in the revision) and others.

Rev. 2:

Zhang et al. investigated the mechanisms controlling the position and direction of lung branching in mice focusing on the early developmental stages. Using complex knockout studies, the authors show that Wnt5a controls branching morphogenesis via non-canonical PCP signaling, specifically Vangl 1/2. Moreover, the authors show that Foxa 2 is also involved in branching regulation. Interestingly, the authors show that perturbation of PCP proteins leads to changes in cellular organization and, specifically, the focal adhesion regulated by FAK. While I find the results highly relevant and that they can advance our understanding of lung morphogenesis, there are significant points that require to be addressed before this work is published. See below:

1. The authors claim that upon PCP perturbation, cytoskeletal components and mechanical cellular properties are impaired. However, the manuscript does not provide any detailed characterization of cellular morphology, measurements of cellular actomyosin properties, or ECM characterization in vivo. Without any more detailed cellular description, the model presented in Figure 4 T, U does not represent the manuscript's findings.

We have added data to show that the levels of phosphorylated Cofilin (p-Cofilin) were increased in *Vangl1/2*-deficient lungs (Fig. S10 in the revision). This suggests that the assembly and disassembly of actin filaments regulated by Cofilin are affected in the mutant lungs.

Moreover, cells derived from *Vangl1/2* knockout lungs allowed us to reveal defects in cellular morphology, the cytoskeleton and focal adhesions (Fig. 3X, 3Y, 3Z, 3A' in the revision).

Finally, we have modified the model in Fig. 4T and 4U (now Fig. 4Z) to more accurately reflect the data presented in this study.

2. The authors should improve the quantification of p-FAK in vivo. As in point above, imaging at the cellular and sub-cellular levels would be essential to understand how p-FAK distribution changes.

We have included images at a higher resolution to better visualize the distribution and intensity of FAK (Fig. 3M, 3N, 3P, 3Q in the revision) and p-FAK (Fig. 3S, 3T, 3V, 3W in the revision). FAK and p-FAK was widely expressed in lung epithelial cells and the signal was concentrated along the apical and basal surface. The p-FAK signal was reduced but the subcellular distribution of p-FAK was unaltered in *Vangl1/2*-deficient lungs. The intensity of FAK and p-FAK was quantified (Fig. 3K in the revision).

Western blot analysis for p-FAK in lysates from *Wnt5a* mutant lungs, *Vangl1/2*-deficient lungs and *Vangl1/2*-deficient cells is provided in Fig. S9 in the revision.

3. It is unclear what the function of Foxa2 is in terms of regulating the morphology of brunching. Again, cellular imaging could help to distinguish between the role of Vangl and Foxa2

Loss of *Foxa1* and *Foxa2* transcription factors has been reported to result in defective branching (Wan et al. *J Biol Chem* 2005 PMID: 15668254). The more severe branching defects in *Wnt5a* mutant lungs than *Vangl1/2* lungs suggest that *Wnt5a* has additional targets besides *Vangl1/2*. Our transcript analysis of mutant lungs implicates *Foxa2* as one of the potential targets of *Wnt5a*. The role of nuclear *Foxa2* in epithelial cells to mediate *Wnt5a* function in lung branching requires future investigation. VANGL2 is expressed in all lung cells and is concentrated at the

apical region of epithelial cells, where VANGL2 expression colocalizes with p-FAK and the actin cytoskeleton (Fig. S5A-5M). We have clarified this point in the revision.

4. For FAK inhibitor experiments, please provide quantification of p-FAK reduction. Also, I can not find the figure of western blot mentioned in the text. Do levels of E-Cadhering also change upon FAK inhibition?

We have provided quantification of p-FAK reduction in lung explants treated with FKA inhibitor (Fig. S12 in the revision).

As mentioned above, Western blot analysis for p-FAK in lysates from *Wnt5a* mutant lungs, *Vangl1/2*-deficient lungs and *Vangl1/2*-deficient cells is provided in Fig. S9 in the revision.

E-Cad levels were reduced upon FAK inhibition. However, reduction in E-Cad levels was similar in lung explants treated with different concentrations of PF-573228.

Minor comments:
1. For figure 1 J, K, L, statistical test is missing. Same for figure 2 P, Q, R,

We have added statistical analysis to Fig. 1J, 1K, 1L and Fig. 2P, 2Q, 2R.

2. Please consider changing the red/green combination to magenta/green

We have changed red/green combination to magenta/green for figures wherein magenta/green can enhance the contrast.

Rev. 3:
In this manuscript, the authors report that Wnt5a expressed in the lung mesenchyme, and to a lesser extent in the lung epithelium, acts through Vangl1/2 mediated PCP to regulate branching morphogenesis during early lung development. While the role of Wnt5a has been report in lung development, the authors made novel findings in this well written short report in the following aspects:

1. Identify the role of Wnt5a-Vangl1/2 PCP signaling in controlling the position and direction of early lung branching.

2. Showing that cytoskeletal reorganization and focal adhesion are regulated by Wnt5a-Vangl PCP in early lung morphogenesis.

3. Wnt5a regulates both PCP and Beta-catenin during lung morphogenesis.
The data are of high quality and nicely presented. Altogether the current study will spur future research in multiple directions.

Minor comments:
1.The authors should quantify p-FAK in Fig. 3E-N. The authors stated that "the levels of p-FAK were significantly reduced in the lung epithelium of mutant lungs, and this conclusion was confirmed by Western blot analysis of lysates from Wnt5a- or Vangl1/2-deficient lungs". I did not see any Western blotting results.

We have included images at a higher resolution to better illustrate a reduction in p-FAK in *Vangl1/2* mutant lungs (Fig. 3S, 3T, 3V, 3W in the revision), which is also quantified (Fig. 3K in the revision). Western blot analysis for p-FAK in lysates from *Wnt5a* mutant lungs, *Vangl1/2*-deficient lungs and *Vangl1/2*-deficient cells is provided in Fig. S9 in the revision.

2. Fig. 3O-R, the authors showed cellular properties of control and Vangl1/2 knockout cells on fibronectin-coated dishes. What are these cells?

Control and *Vangl1/2* knockout cells were derived from control and *Vangl1/2*-deficient lungs collected at 12.5 *dpc*. The procedure of cell derivation is detailed in *Methods*.
