# Supplementary material for: Wnt5a–Vangl1/2 signaling regulates the position and direction of lung branching through the cytoskeleton and focal adhesions
Source: PLoS Biol. 2022 Aug 26;20(8):e3001759. doi: 10.1371/journal.pbio.3001759 (PMC9469998; doi:10.1371/journal.pbio.3001759)
Supplement: Attachment — Submitted filename: Response to Reviewers.pdf [file pbio.3001759.s017.pdf]

Reviewers' comments

Rev. 1:
In their revised manuscript, the authors have newly stained for p-cofilin and shown defects in Vangl1/2 mutant cell morphology, consistent with their model. They have also added a revised model for how branch position could be disrupted due to alterations in mechanical force. However, I still think direct experimental evidence supporting that cytoskeletal and focal adhesion defects are the basis for the mis-localized branch phenotype is lacking.

Wnt5a-Vangl1/2 are well-known to control the cytoskeleton and focal adhesions but how this translates into the observed phenotype remains rather vague and essentially purely speculative, which is my primary concern with this report.

Many of the proofs are quite indirect and the presence of p-FAK in the Wnt5a mutant lungs is still concerning that it may not be the primary effector of the phenotype, as implied in the manuscript title. The lack of a phenotype from isolated Vangl1/2 deletion in either the mesenchyme or epithelium does not clarify this concern. Similarly, the lack of information on whether the incomplete penetrance can be correlated with p-FAK levels leaves the picture confusing.

Overall, I feel the data still do not sufficiently support the main claim of the manuscript.

We appreciate the reviewer's concern that changes in the cytoskeleton and focal adhesions may not fully explain the phenotypes in *Wnt5a/Vangl1/2* mutant lungs. We would like to emphasize that the phenotypic analysis of *Wnt5a/Vangl1/2* mutant lungs establishes a strong foundation for an in-depth analysis of the molecular events due to loss of *Wnt5a/Vangl1/2*. In this regard, our phenotypic and molecular analyses have implicated the cytoskeleton and focal adhesions as mediators of PCP signaling in lung branching. Further investigation is beyond the scope of this study as a Short Report.

A complicated event such as lung branching likely requires the coordination of multiple cellular processes. No single cellular process may be solely responsible for the phenotypes. Nevertheless, our work suggests that the cytoskeleton and focal adhesions contribute significantly to the observed phenotypes.

Essential cellular processes (such as FAK/p-FAK) are controlled by multiple pathways. It is anticipated that loss of one of the regulators compromises but does not eliminates a given cellular process (*e.g.*, a complete loss of p-FAK).

The lack of phenotypes from isolated *Vangl1/2* deletion in either the epithelium or mesenchyme could be a technical limitation in Cre lines and/or represent a complicated interaction between the epithelium and mesenchyme during branching. This issue cannot be resolved in the current study.

While it is difficult to establish a correlation between the incomplete penetrance of phenotypes and p-FAK levels, we did notice a correlation between the appearance of branching defects and reduced p-FAK levels. This further supports our model.

Rev. 2:
Dear Authors,

Thank you for addressing the comments. Levels of phosphorylated Cofilin (p-Cofilin) do not say

much about actin filaments organisation/regulation. However, I agree with the authors that further, more detailed studies focusing on cytoskeletal mechanics are beyond the scope of this paper. Thus, in my opinion, the authors addressed and answered my comments adequately.

As the reviewer pointed out, additional studies are required to further understand the molecular basis of the phenotypes in *Wnt5a/Vangl1/*2 mutant lungs.
